# Number of negative lymph nodes can predict survival of breast cancer patients with four or more positive lymph nodes after postmastectomy radiotherapy

**DOI:** 10.1186/s13014-014-0284-5

**Published:** 2014-12-16

**Authors:** San-Gang Wu, Jia-Yuan Sun, Juan Zhou, Feng-Yan Li, Hao Zhou, Qin Lin, Huan-Xin Lin, Yong Bao, Zhen-Yu He

**Affiliations:** Xiamen Cancer Center, Department of Radiation Oncology, The First Affiliated Hospital of Xiamen University, Xiamen, 361003 People’s Republic of China; Department of Radiation Oncology, Collaborative Innovation Center of Cancer Medicine, State Key Laboratory of Oncology in South China, Sun Yat-sen University Cancer Center, Guangzhou, 510060 People’s Republic of China; Xiamen Cancer Center, Department of Obstetrics and Gynecology, The First Affiliated Hospital of Xiamen University, Xiamen, 361003 People’s Republic of China; Department of Basic Medical Science, Medical College, Xiamen University, Xiamen, 361003 People’s Republic of China

**Keywords:** Breast cancer, Mastectomy, Negative lymph nodes, Radiotherapy

## Abstract

**Background:**

This study was conducted to assess the prognostic value of the number of negative lymph nodes (NLNs) in breast cancer patients with four or more positive lymph nodes after postmastectomy radiotherapy (PMRT).

**Methods:**

This retrospective study examined 605 breast cancer patients with four or more positive lymph nodes who underwent mastectomy. A total of 371 patients underwent PMRT. The prognostic value of the NLN count in patients with and without PMRT was analyzed. The log-rank test was used to compare survival curves, and Cox regression analysis was performed to identify prognostic factors.

**Results:**

The median follow-up was 54 months, and the overall 8-year locoregional recurrence-free survival (LRFS), distant metastasis-free survival (DMFS), disease-free survival (DFS), and overall survival (OS) were 79.8%, 50.0%, 46.8%, and 57.9%, respectively. The optimal cut-off points for NLN count was 12. Univariate analysis showed that the number of NLNs, lymph node ratio (LNR) and pN stage predicted the LRFS of non-PMRT patients (*p* < 0.05 for all). Multivariate analysis showed that the number of NLNs was an independent prognostic factor affecting the LRFS, patients with a higher number of NLNs had a better LRFS (hazard ratio = 0.132, 95% confidence interval = 0.032-0.547, *p* =0.005). LNR and pN stage had no effect on LRFS. PMRT improved the LRFS (*p* < 0.001), DMFS (*p* = 0.018), DFS (*p* = 0.001), and OS (*p* = 0.008) of patients with 12 or fewer NLNs, but it did not any effect on survival of patients with more than 12 NLNs. PMRT improved the regional lymph node recurrence-free survival (*p* < 0.001) but not the chest wall recurrence-free survival (*p* = 0.221) in patients with 12 or fewer NLNs.

**Conclusions:**

The number of NLNs can predict the survival of breast cancer patients with four or more positive lymph nodes after PMRT.

**Electronic supplementary material:**

The online version of this article (doi:10.1186/s13014-014-0284-5) contains supplementary material, which is available to authorized users.

## Background

The purpose of postmastectomy radiotherapy (PMRT) is to improve survival by eliminating potential occult lesions in the chest wall and lymphatic drainage area. The status of axillary lymph nodes is an important factor that affects the choice to use PMRT. PMRT is a standard adjuvant postoperative therapy for patients with four or more positive lymph nodes [1-3]. The local recurrence rate (LRR) of patients with four or more positive lymph nodes who did not receive PMRT was 11.9-59% [4-6]. Thus, about 40% of patients with four or more positive lymph nodes do not benefit from PMRT. At present, there is a trend for oncologists to use individualized treatments for different breast cancer patients. However, it is still difficult to predict which patients with four or more positive lymph nodes will benefit from PMRT.

In breast cancer patients, there is a critical correlation between the status and dissection of axillary lymph nodes, especially with regard to the number of removed lymph nodes (RLNs) [7,8]. The total number of RLNs includes positive lymph nodes and negative lymph nodes (NLNs), so this number may not be a reliable clinical indicator. In addition, due to the different pathological features of lymph nodes, there may be different numbers of occult lesions in the different numbers of NLNs. In theory, removal of more NLNs may reduce the overall risk of occult lesions and thereby improve patient survival. If the number of NLNs is relatively small, the possible presence of occult lesions may increase the LRR. Previous research reported that the number of NLNs might affect the prognosis of breast cancer patients [9,10]. However, the usefulness of the number of NLNs in predicting outcome after PMRT has not yet been reported. We hypothesized that the number of NLNs affects the LRR of breast cancer patients with four or more positive lymph nodes and thereby affects the outcome of PMRT. The current study is a retrospective analysis that investigated the predictive value of the number of NLNs in breast cancer patients with four or more positive axillary lymph nodes after PMRT.

## Materials and methods

### Patients

We retrospectively evaluated 605 breast cancer patients who were received mastectomy at the Sun Yat-sen University Cancer Center between January 1998 and December 2007. All included patients were females who: (*i*) had pathologically confirmed diagnoses of unilateral invasive breast cancer; (*ii*) received mastectomy and axillary lymph node dissection (at least I-II levels) with 10 or more lymph nodes; (*iii*) had stage pT1-4 N2-3 M0 cancer according to the 7th edition of the American Joint Committee on Cancer/Union for International Cancer Control (AJCC/UICC) tumor node metastasis (TNM) staging system; (*iv*) the tumor was completely removed and the margins were negative; (*v*) no neoadjuvant chemotherapy was administered before surgery, and receive at least 4 cycles of postoperative adjuvant chemotherapy; (*vi*) complete immunohistochemistry results including estrogen receptor (ER), progesterone receptor (PR), and human epidermal growth factor receptor 2 (HER2), and endocrine therapy was administered when indicated.

### Clinical and pathological factors and lymph node status

Patient clinicopathological and immunohistochemical factors including age, menstrual status, pT stage, pN stage, NLN count, lymph node ratio (LNR), ER, PR, HER2, breast cancer subtypes (BCS), and PMRT. ER and PR positivity were defined by the presence of more than 1% positive cells based on immunohistochemistry results; HER2 positivity was defined as 3+ or 2+ with confirmation by fluorescence in situ hybridization (FISH). The BCS were not determined according to the criteria developed in the St. Gallen International Breast Cancer Conference because immunohistochemistry results for Ki-67 were not available for some patients [11]. Thus, the categorization of BCS was based on ER, PR, and HER2 status as follows: luminal A (ER+ and/or PR+, and HER2-), luminal B (ER+ and/or PR+, and HER2+), HER-2 + (ER-, PR-, and HER2+), and triple negative (TN) (ER-, PR-, and HER2-).

We identified the pT/pN stages according to the 7th edition of the AJCC/UICC TNM staging system. Stage pN2 was defined by metastases in 4–9 lymph nodes and stage pN3 by metastases in 10 or more lymph nodes. The number of removed NLNs was obtained by subtracting the number of positive lymph nodes from the total number of removed axillary lymph nodes. LNR classifications were based on the report by Vinh-Hung et al. [12] and our previously reported [13,14]. Patients were classified into 3 groups with LNR 0.01-0.20, LNR 0.21 - 0.65, and LNR > 0.65.

### Treatment

Adjuvant chemotherapy was administered to all patients for a median of 6 cycles (range: 4–8 cycles), with 29 patients (4.8%) receiving a regimen consisting of cyclophosphamide, methotrexate, and 5-fluorouracil (CMF), and 576 (95.2%) receiving regimens with anthracycline and/or taxane. All patients were recommended for PMRT, but the patient made the final decision over whether to proceed. Of the 234 patients (38.7%) did not receive PMRT due to economic and social factors. A total of 371 patients (61.3%) received PMRT within 6 months after surgery. The PMRT was delivered mainly to the ipsilateral chest wall, supra- and infra-clavicular lymph node regions. The total radiation dose was 46–50 Gy in 23–25 fractions. The chest wall was treated with 6 MV X-ray with opposed tangential fields or 6–9 Mev electron beam, with the use of tissue compensation membrane of 0.5–1 cm when needed. Single-field irradiation was performed for the supra- and infra-clavicular lymph drainage regions with 6 MV X-ray combined with 12–15 Mev. Patients with tumours positive for the ER or PR (or both) received endocrine therapy, mainly with tamoxifen or aromatase inhibitors.

### Follow-up and survival endpoints

Follow-up was performed once every 3 to 6 months. Locoregional recurrence-free survival (LRFS) was the primary endpoint. Distant metastasis-free survival (DMFS), disease free survival (DFS), and overall survival (OS) were the secondary endpoints. Locoregional recurrence refers to pathologically confirmed recurrence at the ipsilateral chest wall, supraclavicular and subclavian lymph nodes, axillary lymph nodes, or internal mammary lymph nodes. Distant metastasis refers to recurrence at a site distant from the primary cancer based on two imaging examinations or by pathologic assessment. OS was calculated as the time from the date of diagnosis to the date of death from any cause or to the date of last follow-up.

### Statistical analysis

All data were analyzed using the SPSS statistical software package (version 16.0; IBM Corporation, Armonk, NY, USA). The χ^2^ and Fisher’s exact probability tests were used to analyze differences in the qualitative data. Cut-off point analyses were then performed to determine whether there was a cut-off NLN number that was related to the greatest difference in LRFS. The optimum cut-off point for the NLNs was determined by use of the receiver operating characteristic (ROC) curve. Calculation of survival rates were plotted by the Kaplan-Meier method and compared using the log-rank test. To determine the effect of the number of NLNs on LRFS, we performed univariate and multivariate Cox regression model analysis. The variables with *p*-values less than 0.05 by univariate analyses were included in the multivariate analyses. A *p*-value less than 0.05 was considered statistically significant.

## Results

### Patient clinicopathologic data and status of lymph node dissection

A total of 605 patients were enrolled and 371 patients (61.3%) underwent PMRT. Table 1 shows the clinicopathologic data of the enrolled patients. Except for the greater use of PMRT in patients with pN3 disease, there were no significant differences between the PMRT and non-PMRT groups.

**Table 1:** **Characteristics of breast cancer patients who received and did not receive postmastectomy radiotherapy**

**Characteristic**	**n**	**Without PMRT**	**With PMRT**	***p***
		**(n = 234) (%)**	**(n = 371) (%)**	
Age, years				
<35	64	20 (8.6)	44 (11.9)	0.197
≥35	541	214 (91.4)	327 (88.1)	
Menopausal status				
Premenopausal	400	153 (65.4)	247 (66.6)	0.763
Postmenopausal	205	81 (34.6)	124 (33.4)	
Tumor size				
T1-T2	471	189 (80.8)	282 (76.0)	0.170
T3-T4	134	45 (19.2)	89 (24.0)	
Nodal stage				
N2	321	139 (59.4)	182 (49.1)	0.013 ^*^
N3	284	95 (40.6)	189 (50.9)	
ER status				
Negative	285	112 (47.9)	173 (46.6)	0.767
Positive	320	122 (52.1)	198 (53.4)	
PR status				
Negative	229	92 (39.3)	137 (36.9)	0.555
Positive	376	142 (60.7)	234 (63.1)	
HER2 status				
Negative	376	136 (58.1)	240 (64.7)	0.105
Positive	229	98 (41.9)	131 (35.3)	
Breast cancer subtype				
Luminal A	295	107 (45.7)	188 (50.7)	0.368
Luminal B	130	53 (22.6)	77 (20.8)	
HER2 positive	99	45 (19.3)	54 (14.5)	
Triple negative	81	29 (12.4)	52 (14.0)	
Number of NLNs				
0-12	477	180 (76.9)	297 (80.0)	0.358
12-39	128	54 (23.1)	74 (20.0)	
LNR				
< 0.20	42	29 (12.4)	13 (3.5)	< 0.001^*^
0.21-0.65	337	131 (56.0)	206 (55.5)	
> 0.65	226	74 (31.6)	152 (41.0)	

PMRT, post-mastectomy radiotherapy; ER, estrogen receptor; PR, progesterone receptor; HER2, human epidermal growth factor receptor-2; NLNs, negative lymph nodes; LNR, lymph node ratio.

^*^ *p* < 0.05 indicates a significant difference.

The median number of RLNs in the entire cohort was 18 (range: 10–73); the median number in the non-PMRT group and the PMRT group were 18 (range: 10–45) and 18 (range: 10–73), respectively. The median number of NLNs in the non-PMRT group and the PMRT group was 8 (25th percentile: 5, 75th percentile: 12; range, 0–39) and 7 (25th percentile: 4, 75th percentile: 12; range: 0–36), respectively.

### Survival

The median follow-up time of all patients was 54 months (range: 6–138 months). A total of 91 patients had locoregional recurrence, and the 5-year LRFS and 8-year LRFS were 83.3% and 79.8%, respectively. Two hundred forty-seven patients had distant metastases and the 5-year DMFS and 8-year DMFS were 58.3% and 50.0%, respectively. The 5-year DFS and 8-year DFS were 55.1% and 46.8%, respectively. One hundred ninety-six patients died and the 5-year OS and 8-year OS of these patients were 69.9% and 57.9%, respectively.

### Identification of optimal cut-off points of NLNs

**Figure 1:**
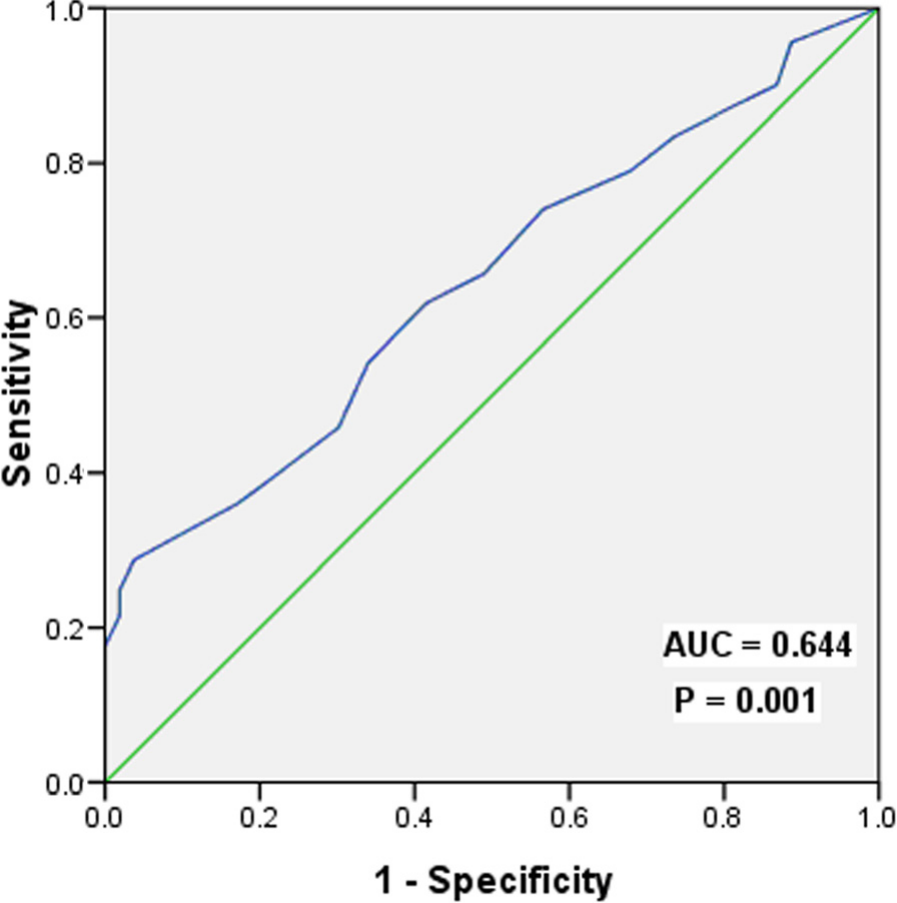
**The receiver operating characteristic curve of the optimum cut-off point for the NLNs.**

The optimal cut-off points of NLNs were analyzed for non-PMRT patients using ROC curve. The results showed that 12 was the optimal cut-off point for NLNs (Area Under roc Curve = 0.644, *p* = 0.001) (Figure 1). Therefore, the optimal cutoff value of 12 was validated as a prognostic factor for analysis of the clinical effect of the number of NLNs.

The NLN count was associated with pT stage (*p* = 0.003), pN stage (*p* < 0.001), and LNR (*p* < 0.001), but was not associated with age, menstrual status, ER status, PR status, HER2 status, and BCS (all, *p* > 0.05) (Additional file 1: Table S1).

### Analysis of prognostic factors affecting LRFS in patients without PMRT

Univariate analysis of non-PMRT patients showed that the NLN count, LNR, menstruation status, pT stage, and pN stage were prognostic factors that affected LRFS (*p* < 0.05) (Table 2).

**Table 2:** **Univariate and multivariate analysis of factors associated with locoregional recurrence free survival in patients without postmastectomy radiotherapy**

**Characteristic**	**Univariate analysis**			**Multivariate analyses**		
	**HR**	**95% CI**	***p***	**HR**	**95% CI**	***p***
Age, years (<35 vs. ≥35)	0.707	0.302-1.656	0.425	—	—	—
Menopausal status (premenopausal vs. postmenopausal)	0.473	0.244-0.919	0.027^*^	0.494	0.254-0.961	0.038^*^
Tumor size (T3-T4 vs. T1-T2)	2.708	1.529-4.797	0.001^*^	2.193	1.231-3.909	0.008^*^
Nodal stage (N3 vs. N2)	1.991	1.160-3.418	0.012^*^	1.577	0.762-3.264	0.219
ER status (positive vs. negative)	0.891	0.516-1.530	0.676	—	—	—
PR status (positive vs. negative)	1.278	0.700-2.222	0.452	—	—	—
HER2 status (positive vs. negative)	1.032	0.595-1.790	0.912	—	—	—
Breast cancer subtype						
(luminal B vs. luminal A)	1.309	0.676-2.533	0.424	—	—	—
(HER2 positive vs. luminal A)	0.855	0.368-1.988	0.716	—	—	—
(triple negative vs. luminal A)	1.409	0.632-3.137	0.402	—	—	—
Number of NLNs, n (12–39 vs. 0–12)	0.111	0.027-0.458	0.002	0.132	0.032-0.547	0.005
LNR						
(0.21-0.65 vs. < 0.20)	3.608	0.859-15.152	0.080	2.950	0.646-13.465	0.163
(> 0.65 vs. < 0.20)	7.473	1.759-31.755	0.006*	3.376	0.773-14.750	0.106

ER, estrogen receptor; PR, progesterone receptor; Her-2, human epidermal growth factor receptor-2; NLNs, negative lymph nodes; LNR, lymph nodes ratio.

^*^ *p* < 0.05 indicates a significant difference.

Multivariate analysis showed that the number of NLNs was an independent prognostic factor for LRFS. Patients with a higher number of NLNs had a better LRFS (hazard ratio [HR]: 0.132, 95% confidence interval [CI]: 0.032-0.547, *p* = 0.005), but pN stage and LNR did not affect LRFS (*p* > 0.05). In addition, menopausal status and pT stage were also independent risk factors for LRFS (*p* < 0.05 for both) (Table 2).

**Figure 2:**
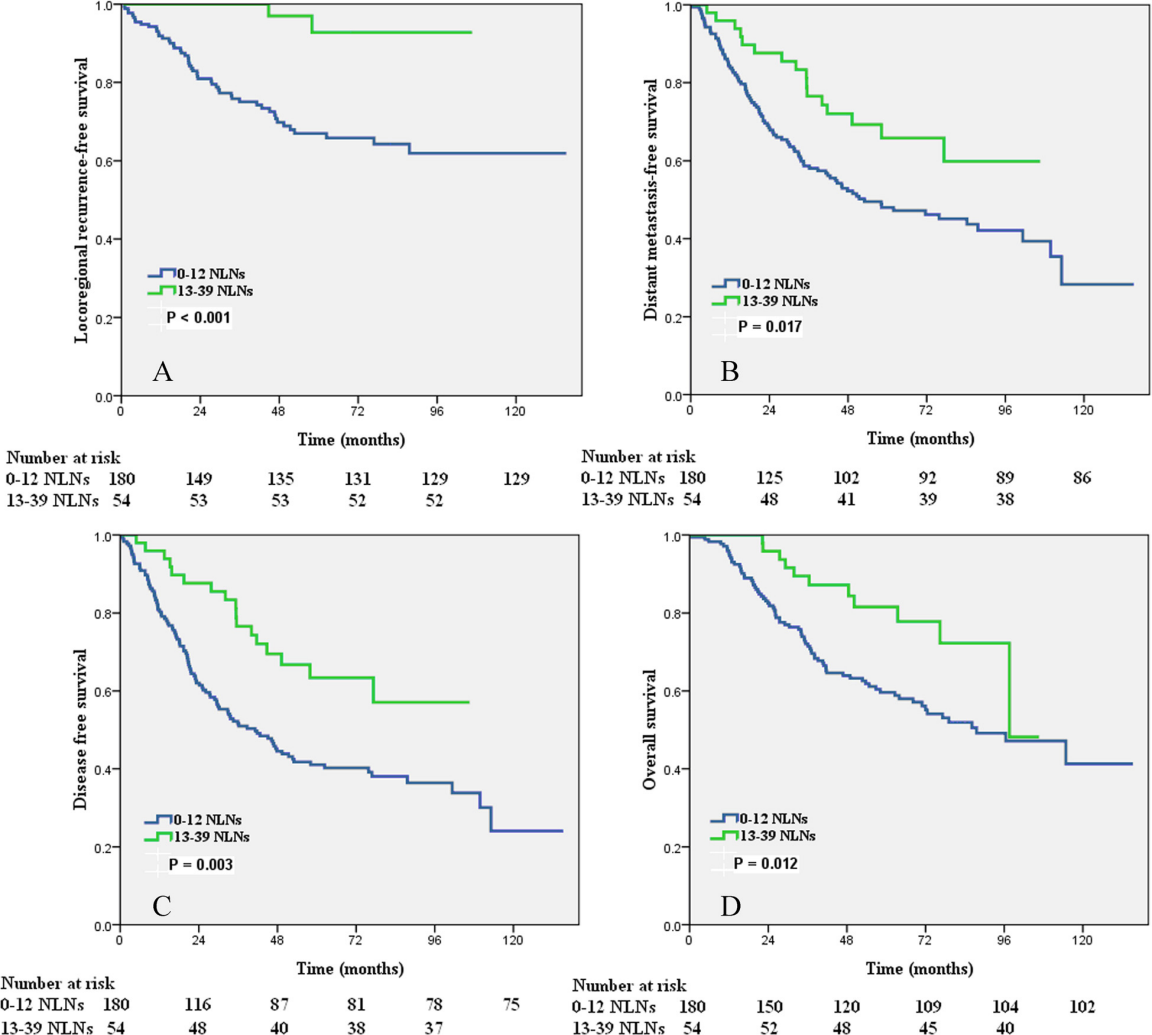
**Impact of the number of negative lymph nodes on the locoregional recurrence-free survival (A), distant metastases-free survival (B), disease-free survival (C), and overall survival (D) of patients with and without postmastectomy radiotherapy.**

In patients without PMRT, a higher number of NLNs was correlated with a better LRFS (*p* < 0.001), DMFS (*p* = 0.017), DFS (*p* = 0.003), and OS (*p* = 0.012) using the log-rank test (Figure 2).

### Prognostic value of the number of NLNs in patients undergoing PMRT

Compared to non-PMRT patients, PMRT patients had better outcomes, with significantly improved LRFS (*p* < 0.001), DMFS (*p* = 0.012), DFS (*p* = 0.001), and OS (*p* = 0.013)..

**Figure 3:**
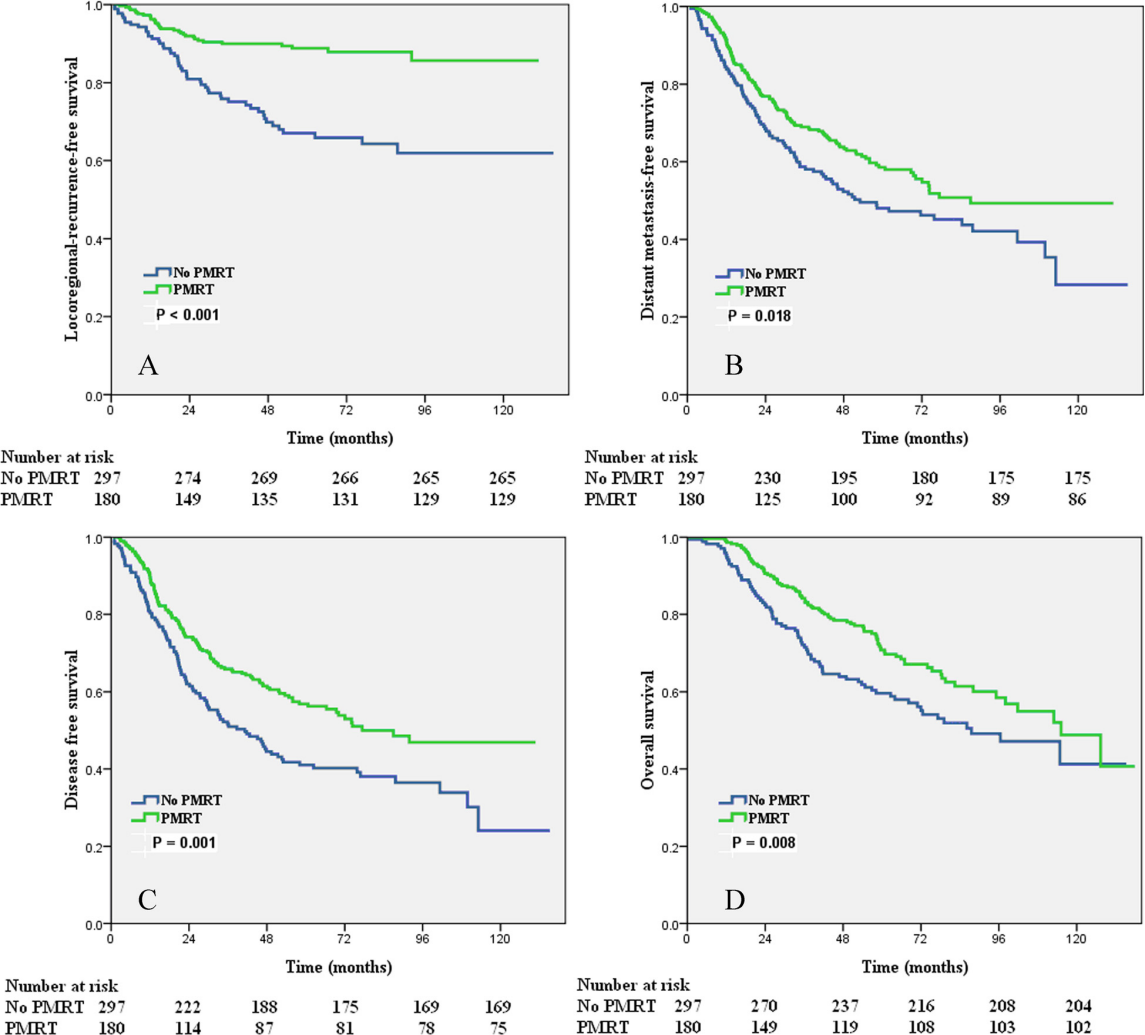
**Impact of postmastectomy radiotherapy on locoregional recurrence-free survival (A), distant metastases-free survival (B), disease-free survival (C), and overall survival (D) of patients with 12 or fewer NLNs.**

Subgroup analysis showed that different numbers of NLNs could predict the outcome in patients received PMRT. PMRT improved the LRFS (*p* < 0.001), DMFS (*p* = 0.018), DFS (*p* = 0.001), and OS (*p* = 0.008) of patients with 12 or fewer NLNs. However, PMRT had no effect on LRFS (*p* = 0.500), DMFS (*p* = 0.204), DFS (*p* = 0.199), and OS (*p* = 0.653) of patients with more than 12 NLNs (Table 3 and Figure 3).

**Table 3:** **Impact of the number of negative lymph nodes on locoregional recurrence-free survival in patients with and without postmastectomy radiotherapy**

**Survival endpoint**	**0-12 NLNs (%)**			**13-39 NLNs (%)**		
	**Without PMRT**	**With PMRT**	***p***	**Without PMRT**	**With PMRT**	***p***
LRFS (8-year)	61.9	85.6	<0.001^*^	92.8	92.1	0.500
DMFS (8-year)	42.1	49.3	0.018^*^	59.9	66.2	0.204
DFS (8-year)	36.5	46.9	0.001^*^	57.1	42.0	0.199
OS (8-year)	49.1	58.5	0.008^*^	72.3	68.2	0.653

PMRT, postmastectomy radiotherapy; NLNs, negative lymph nodes; LRFS, locoregional-recurrence-free survival; DMFS, distant metastasis-free survival; DFS, disease free survival; OS, overall survival.

^*^ *p* < 0.05 indicates a significant difference.

We examined the prognostic effect of the number of NLNs according to local (chest wall) or regional (regional lymph node) recurrences in patients with and without PMRT. In patients with 12 or fewer NLNs, PMRT did not improved the chest wall recurrence, the 8-year chest wall recurrence-free survival was 84.7% and 89.8%, respectively (*p* = 0.221). However, PMRT improved the regional recurrence with the 8-year regional recurrence-free survival was 90.1% and 70.1%, respectively (*p* < 0.001). In patients with more than 12 NLNs, PMRT without any effect on survival of chest wall recurrence (*p* = 0.803) and regional recurrence (*p =* 0.331).

## Discussion

In the present study, we investigated the prognostic value of the number of NLNs in breast cancer patients with four or more positive lymph nodes. The results indicate that the number of NLNs can be used to predict the outcome of PMRT in breast cancer patients.

Total number of RLNs include positive lymph nodes, so use of this number may only have limited prognostic value in identification of breast cancer patients that would benefit from PMRT. Thus, Karlsson et al. found that the number of NLNs is an independent risk factor for LRFS; the LRR of patients with 10 or more NLNs was significantly lower than that of patients with fewer than 10 NLNs, and the difference was especially significant in patients with positive lymph nodes [9]. Kuru reported better OS of patients with more than 15 NLNs [10]. We used Cox multivariate analysis, with NLN count, LNR, and pN stage as covariates, and found that the number of NLNs was an independent prognostic factor for LRFS, but that LNR and pN stage had no significant effect on prognosis. These results suggest that the number of NLNs may be a better indicator of axillary lymph node status in breast cancer patients.

At present, the mechanism underlying why the number of NLNs can be used to predict the survival of breast cancer patients is still unclear. However, previous studies showed that this relationship might be explained by “stage migration” or by the host’s immune response to tumor cells and the molecular biology of tumor cells [15-17]. For example, a study of colorectal cancer patients found that the lymphocyte response to the tumor was related to patient survival [18].

The LRR of breast cancer is an important factor used to guide the selection of PMRT. The status of axillary lymph nodes has been always one of the main factors used to guide selection of PMRT. PMRT remains the standard therapy for breast cancer patients who have four or more positive lymph nodes. PMRT can eliminate residual occult lesions in the ipsilateral chest wall and lymph drainage area, and thereby improve survival. PMRT improves the prognosis of breast cancer patients with 4 or more positive axillary lymph nodes [19]. Our subgroup analysis showed that the LRR was relatively low in patients with a higher number of NLNs, and that PMRT provided no obvious benefit for these patients. In addition, we also found that PMRT improved locoregional control by reducing nodal recurrences but without any effect on local control in the chest wall in patients with a fewer number of NLNs. This supports our hypothesis that patients with fewer NLNs may have more residual lesions, which increases the rate of locoregional recurrence. However, for patients with more NLNs, surgery can reduce the number of residual lesions, and these patients do not benefit significantly from PMRT.

The American College of Surgeons Oncology Group (ACOSOG) Z0011 trial reported that axillary lymph node dissection did not affect the local recurrence and survival rates of patients without positive sentinel lymph nodes or with 1–2 positive sentinel lymph nodes after breast-conserving therapy [20,21]. Thus, axillary lymph node dissection is unnecessary in these patients [20,21]. However, the St. Gallen International Breast Cancer Conference (2013) reported that axillary lymph node dissection was necessary for cases who did not receive radiotherapy and for those with 3 or more metastases in the sentinel lymph nodes [11]. Thus, the status of axillary lymph nodes is an important indicator that affects the prognosis of breast cancer patients and is useful in guiding therapy. In the present study, the number of NLNs significantly affected prognosis and could be used to predict the outcome of radiotherapy. Therefore, when the status of axillary lymph nodes can be accurately determined, axillary lymph node dissection is a very important procedure for patients with positive axillary lymph nodes. In particular axillary lymph node dissection can reduce the risk of occult lesions, allows some patients to omit radiotherapy and thereby avoid radiation injury [22-24], and thereby improves patient quality of life and prolongs survival.

We need to recognize the limitations of the present study. First, this study is a single-center retrospective study and is not representative of the breast cancer population at large. In addition, the optimal number of NLNs in present study is different from the number in other studies. This may be related to differences in the clinical characteristics of the patient populations, use of different surgical procedures, and analysis by different statistical method. However, based on the relationship between the number of NLNs and host immune response [16,17], sufficient attention should be paid to differences in individual patient immune status. Ultimately, multicenter prospective studies should be performed to verify the value of the number of NLNs in breast cancer patients to determine the most appropriate cut-off point. Finally, patients enrolled in the current study did not undergo trastuzumab therapy. Recent studies reported that trastuzumab reduces the rate of locoregional recurrence [25,26].

## Conclusions

In conclusion, the results of the present study show that the number of NLNs is a prognostic factor for LRFS in breast cancer patients with four or more positive lymph nodes after mastectomy. Patients with more NLNs have lower LRR, and the number of NLNs can predict the outcome of PMRT. PMRT might represent for certain patients with a lower number of NLNs, but not in patients with a higher number of NLNs. Future prospective studies are needed to confirm these results.

## Additional file

Additional file 1: Table S1. Characteristics of breast cancer patients according to different number of negative lymph nodes in patients without postmastectomy radiotherapy.

## Abbreviations

PMRT: Post-mastectomy radiotherapy
LRR: Local recurrence rate
RLNs: Removed lymph nodes
NLNs: Negative lymph nodes
LNR: Lymph node ratio
ER: Estrogen receptor
PR: Progesterone receptor
HER2: Human epidermal growth factor receptor 2
TN: Triple negative
BCS: Breast cancer subtpye
FISH: Fluorescence in situ hybridization
AJCC/UICC: American Joint Committee on Cancer/Union for International Cancer Control
TNM: Tumor node metastasis
LRFS: Locoregional recurrence-free survival
DMFS: Distant metastasis-free survival
DFS: Disease free survival
OS: Overall survival
ACOSOG: American College of Surgeons Oncology Group
