# Supplementary material for: Number of negative lymph nodes can predict survival of breast cancer patients with four or more positive lymph nodes after postmastectomy radiotherapy
Source: Radiat Oncol. 2014 Dec 16;9:284. doi: 10.1186/s13014-014-0284-5 (PMC4278342; doi:10.1186/s13014-014-0284-5)
Supplement: Additional file 1: Table S1. — Characteristics of breast cancer patients according to different number of negative lymph nodes in patients without postmastectomy radiotherapy. [file 13014_2014_284_MOESM1_ESM.doc]

**Additional file 1: Table S1. Characteristics of breast cancer patients according to different number of negative lymph nodes in patients without postmastectomy radiotherapy.**

| **Characteristic** | **n** | **0-12 NLNs**  **(n=180) (%)** | **13-39 NLNs (n=54) (%)** | ***p*** |
| --- | --- | --- | --- | --- |
| Age, years |  |  |  |  |
| <35 | 20 | 14 (7.8) | 6 (11.1) | 0.442 |
| ≥35 | 214 | 166 (92.2) | 48 (88.9) |  |
| Menopausal status |  |  |  |  |
| Premenopausal | 153 | 121 (67.2) | 32 (59.3) | 0.281 |
| Postmenopausal | 81 | 59 (32.8) | 22 (40.7) |  |
| Tumor size |  |  |  |  |
| T1-T2 | 189 | 138 (76.7) | 51 (94.4) | 0.003 ^*^ |
| T3-T4 | 45 | 42 (23.3) | 3 (5.6) |  |
| Nodal stage |  |  |  |  |
| N2 | 139 | 95 (52.8) | 44 (81.5) | < 0.001 ^*^ |
| N3 | 95 | 85 (47.2) | 10 (18.5) |  |
| ER status |  |  |  |  |
| Negative | 112 | 85 (47.2) | 27 (50.0) | 0.720 |
| Positive | 122 | 95 (52.8) | 27 (50.0) |  |
| PR status |  |  |  |  |
| Negative | 92 | 68 (37.8) | 24 (44.4) | 0.379 |
| Positive | 142 | 112 (62.2) | 30 (55.6) |  |
| HER2 status |  |  |  |  |
| Negative | 136 | 104 (57.8) | 32 (59.3) | 0.847 |
| Positive | 98 | 76 (42.2) | 22 (40.7) |  |
| Breast cancer subtype |  |  |  |  |
| Luminal A | 107 | 82 (45.6) | 25 (46.2) | 0.972 |
| Luminal B | 53 | 42 (23.3) | 11 (20.4) |  |
| Her2 positive | 45 | 34 (18.9) | 11 (20.4) |  |
| Triple negative | 29 | 22 (12.2) | 7 (13.0) |  |
| LNR |  |  |  |  |
| < 0.20 | 29 | 8 (4.4) | 21 (38.9) | < 0.001 ^*^ |
| 0.21-0.65 | 131 | 98 (54.4) | 33 (61.1) |  |
| > 0.65 | 74 | 74 (41.2) | 0 (0) |  |

PMRT, post-mastectomy radiotherapy; ER, estrogen receptor; PR, progesterone receptor; Her-2, human epidermal growth factor receptor-2; NLNs, negative lymph nodes; LNR, lymph node ratio.

^*^*p* < 0.05 indicates a significant difference.
